# Exploring the decision-making process of female genital cosmetic procedures in Iranian women and constructing and validating a results-based logic model for a healthy public policy: a study protocol

**DOI:** 10.1186/s12978-024-01788-z

**Published:** 2024-04-08

**Authors:** Elham Azmoude, Samira Ebrahimzadeh Zagami, Elahe Hooshmand, Elham Taheri, Nahid Jahani Shoorab

**Affiliations:** 1 Student Research Committee, Mashhad University of Medical Sciences, Mashhad, Iran; 2 Nursing and Midwifery Care Research Centre, Mashhad University of Medical Sciences, Mashhad, Iran; 3 Department of Midwifery, School of Nursing and Midwifery, Mashhad University of Medical Sciences, Mashhad, Iran; 4 Social Determinants of Health Research Center, Mashhad University of Medical Sciences, Mashhad, Iran; 5 Department of Clinical Psychology, School of Medicine, Mashhad University of Medical Sciences, Mashhad, Iran

**Keywords:** Female genital cosmetic procedures, Grounded theory, Logic model, Nominal group technique

## Abstract

**Background:**

Female genital cosmetic procedures have grown rapidly in most parts of the world. Professional organizations have issued warnings about the complications and long-term consequences of these practices. To be able to adopt the right health policies, it is necessary to know why women decide to perform these procedures. Therefore, the present study will be aim to discover the decision-making process involved in performing female genital cosmetic procedures for Iranian women and construct and validate a results-based logic model for healthy public policy.

**Methods:**

The present study was conducted in three phases. In the initial phase, a qualitative study will be conducted with the Corbin and Strauss ground theory approach. The participants in the study will be healthy women who desire or have undergone female genital cosmetic procedures without medical indications. In this phase, purposive and theoretical sampling will guide recruitment and data collection. The data will be collected via semi-structured interviews, field notes and observations of individual interactions. The data will be analysed using the approach of Corbin and Strauss (2015). MAXQDA 2007 software was used for managing the process of data analysis. In the second phase, the development of a results-based logic model for a healthy public policy is performed based on the findings of the first phase of the study, interviews with key informants and a review of the results of the literature in this field. Finally, validation of the designed program will be performed by the nominal group technique with the presence of a group of experts in the third phase.

**Discussion:**

The findings of this study, by identifying women’s main concerns related to the studied phenomenon, the existing context, participants’ reactions and the consequences of the adopted reactions, can be very important in designing a program that fits Iran’s cultural characteristics. In this research, a program using a logical model will be presented that is suitable for policymakers, planners and healthcare service providers to be implemented in the social-cultural context of the study.

## Abstract (plain language summary)

Female genital cosmetic procedures refer to a group of cosmetic procedures that change the structure and healthy appearance of the female external genitalia to improve sexual performance or body image. The desire to perform these techniques has become popular in most parts of the world. However, scientific societies have warned about the efficiency, effectiveness and side effects of these techniques. According to these points, the present study aims to discover the decision-making process of performing FGCPs for Iranian women and to construct and validate a program for healthy public policy. This study will be performed in three stages. First, a qualitative study and interviews with healthy women who desire or have undergone female genital cosmetic procedures will be performed. In the following, based on the findings of the first stage, interviews with key informants and a review of literature, a program will be presented to reduce or prevent these procedures, and then this program will be validated. Using the designed program, healthcare practitioners will be able to provide women with more effective advice and guidance to make correct and informed decisions. In addition, this program will enable planners and policymakers to take steps to reduce the demand for these actions and make informed decisions by women by changing and adjusting the conditions and context.

## Background

The use of surgical knives on women’s bodies has a long history, ranging from unnecessary episiotomy and unnecessary caesarean section to female circumcision and, recently, female genital cosmetic procedures (FGCPs) [1]. FGCPs include various surgical and nonsurgical techniques, which are often performed to achieve a better genital appearance and improve women’s sexual performance without any medical indications. Labiaplasty, mons pubis liposuction, bleaching, and G-spot augmentation are among these procedures [2].

Despite insufficient evidence about the effectiveness and side effects of these techniques, the rate of these procedures has been increasing worldwide and in most Western and Eastern countries [3]. In 2016, the International Society of Cosmetic Surgery registered 138,033 cases of labiaplasty worldwide, which was a 46% increase compared to 2015, and its growth rate was much greater than that of other cosmetic surgeries [4]. The statistics published in the United States in 2018 also indicate 12,756 cases of labiaplasty, which is a 53% increase compared to the last 5 years [5]. A similar increase has been reported in Canada, some European countries, Australia, Brazil and parts of Asia from 2001 to 2016 [6, 7].

Based on the available data, although exact statistics for performing these procedures in Iran are not available, it is thought that Iran is a country with high statistics for FGCPs, which has become a social concern in addition to a medical concern [8].

According to the opinion of experts, some FGCPs are similar to female genital mutilation in terms of applied anatomical changes as well as direct and indirect social pressures that lead women to perform these procedures [9]. On the other hand, public concern about the ethical foundations, safety and efficiency of these procedures has increased [10]. Despite the many goals that are expressed to perform these practices, there is limited evidence to support their efficiency and effectiveness in improving the perception of women’s bodies and sexual performance [11]. In this context, the American College of Obstetricians and Gynecologists (ACOG) points out that due to the limited information on the safety and effectiveness of genital cosmetic surgeries and the existence of potential complications does not support their use [12].

According to the literature, the desire to change the appearance of the genitals may be based on incorrect assumptions about the natural dimensions of the body. Accordingly, some studies attribute the demand for FGCPs to women’s desire to standardize the appearance of their genitalia, with the labia minora being narrow and not visible and the vaginal opening appearing too tight [13, 14]. The decision to perform FGCPs is based on culture and is also affected by the development of internet pornography, the normalization of pubic hair removal and the uniform display of the female reproductive system on websites, magazines and journals [14, 15]. Exposure to images of modified genitalia may change women’s perception of what is normal or desirable and influence their decision to undergo labiaplasty [12]. For example, in the case‒control study conducted by Sharp et al. (2016), the strongest motivation for Australian women to undergo labiaplasty was concern about their genital appearance [16].

One of the important issues in the pathology of the psychological and economic burden of these practices is knowing why women decide to perform these techniques. Most of the studies on the causes and factors related to women’s decision-making have been conducted in Western countries with quantitative and cross-sectional designs and based on a positivist paradigm [16–18]. Moreover, a deep understanding of why and how complex phenomena such as the tendency towards FGCPs can be achieved through qualitative studies [19]. This type of study is important because women’s tendency toward FGCPs is rooted in cultural and psychosocial factors, and women’s decision-making takes place during a multidimensional and complex interactive process [7, 19]. In this regard, Locatelli et al. (2017) conducted a qualitative study with a grounded theory approach to explain the decision-making process and willingness to perform cosmetic surgeries (including general and genital cosmetic surgeries). Based on the results of the in-depth interviews, the two main factors identified that can lead to the decision to undergo cosmetic surgery include recent life events and psychological needs [19].

Among the various methods of qualitative research, grounded theory is used if the perception of the phenomenon in the context of society and the study of social processes embedded in human interactions are desired [20]. Therefore, explaining the decision-making process of FGCPs through a qualitative study with ground theory seems necessary due to the unavailability of a similar study.

The results of qualitative studies are very suitable for use in the design of health programs because they are based on the views of participants and beneficiaries. Among these methods, the use of the ground theory method, due to the provision of deep and comprehensive data about the various dimensions of phenomena, can be a very suitable methodology for planning effective programs [21]. Therefore, studies such as this one will be more effective when the results are used in the direction of policy-making and the formulation of health programs. One of the appropriate tools for designing health programs, especially in the field of reproductive health, is the logical model [22]. A logic model is a programming tool used to visualize the inputs and activities of a program and the expected results of the program. Logic models provide a framework that allows planners, evaluators, and other stakeholders to link program processes to program outcomes [23].

The results of ground theory studies can be very important in designing a suitable program by identifying the concerns of women about the studied phenomenon, context, individual reactions and consequences of the adopted reactions [21]. In this study, the emergence of the process in which women decide to perform FGCPs can be effective in the design of health programs. Therefore, the researchers will try to present a program logic model for healthy public policy based on the results of the qualitative section and using the points of view of key informants. According to the mentioned notes, this protocol will be designed to discover the decision-making process of performing FGCPs in Iranian women and construct and validate a results-based logic model for healthy public policy.

### Study aims

- 1- The discovery of the decision-making process for performing FGCPs among women.
- 2- Constructing a results-based logic model for a healthy public policy.
- 3- Validation of a results-based logic model for a healthy public policy using a nominal group technique.

### The main research questions

How is the FGCPS decision-making process formed for women?

## Methods/design

### The first phase: a qualitative study

#### Design

In this phase, a qualitative study will be conducted based on the grounded theory approach to discover the decision-making process for performing FGCPs in Iranian women. Grounded theory, as an inductive qualitative approach, is a systematic approach for collecting and analysing qualitative data to construct theories that are grounded in the data [20].

#### Study setting

This study will be conducted in Mashhad, one of the largest cities in Iran and the capital of Khorasan-e Razavi Province. Participants will be recruited from healthcare centers, governmental and private hospitals and gynecology clinics. The most common FGCPs currently performed in this city are labiaplasty, perineoplasty, vaginoplasty, HIFU vaginal rejuvenation and vaginal carboxytherapy.

#### Inclusion criteria

In this study, the participants will be healthy women who desire or have undergone FGCPs without medical indications (such as stage 3 or 4 uterine prolapse or vaginismus). Having agreed to participate in the study, being Iranian, and having less than 5 years since FGCPs were among the other eligibility criteria for inclusion in this study. A history of psychological illness or the use of psychological drugs will exclude women from this study.

Participants will be identified using the documentation available in health centers, hospitals, and clinics. In addition, access to participants will be achieved by installing information posters in health centers and clinics.

#### Data collection method

The data will be collected through interviews, field notes and observations of individual interactions in the research environment. Semi-structured face-to-face in-depth interviews will be the main method of data collection. An interview guide with open-ended and possible follow-up questions will be designed to explore the experiences of each participant. The interview will begin with an open and general question such as “Please tell me about the first time you thought of using the procedure”. During the interviews, the questions gradually will be become more specific and focused. For example, the following question will be asked: “After taking action to use these procedures, what changes did you experience in your life?“. In addition, probing questions will be used if needed.

Data collection in this phase will begin after the research objectives are explained by the researchers and the written consent form is completed by the participants. The interviews will be conducted individually in private settings chosen by participants (e.g., their home or the public library or hospital).

Examples of the interview questions are as follows:

- What made you think of FGCPs?
- Can you talk about your experience of using FGCPs?
- With whom did you discuss your decision to perform FGCPs, and how did you see their reaction?
- Can you explain to me what happened that made you make the final decision to do FGCPs?
- What changes did you experience in your life after FGCPs?

#### Sample size and sampling method

In this study, sampling continued until data saturation. This means that the data collection will continue until no new dimensions or features are added to the emerging categories and no new relationships appear between the categories [20, 24].

In this study, purposive and theoretical sampling will guide recruitment and data collection. First, women who could express their in-depth feelings and experiences regarding FGCPs will be required purposefully based on the inclusion criteria.

Purposive sampling will be used to select participants by adopting a maximum variation strategy based on age, marital status, education, social class, occupation, motivation to perform the procedure, type of procedures, time elapsed from the procedures, and decision stage (including precontemplation, contemplation, preparation, action, maintenance and even people who have given up).

In addition, theoretical sampling will also be used to recruit additional participants to guide the depth of the theory. Theoretical sampling is a process of collecting data that helps researchers create a theory that is grounded in the data [20, 24].

Along with theoretical sampling and to complete the theory, it may be necessary to include influential individuals in the decision-making process for FGCPs, such as health and medical professionals (such as reproductive health policymakers, gynecologists, midwives, plastic and cosmetic surgeons, psychologists, psychologists and nurses, etc.), women’s family members (for example, spouses) or even women who are not applying for FGCPs.

#### Data analysis

In this study, the data collection and analysis process will be performed continuously and simultaneously from the beginning of the research. In this study, the approach of Corbin and Strauss (2015) will be used for data analysis. The approach of Corbin and Strauss 2015 involves six steps, including analysing data for concepts, developing the concepts in terms of their properties and dimensions, analysing data for context, bringing the process into the analysis, and integrating categories.

In this study, according to Corbin and Strauss, analytical tools such as using questions (sensitizing questions, theory questions), comparing (continuous comparison, theoretical comparison), the Flip-flop technique and waving the red flag will be used used to analyse the data in terms of context, process and composition of categories [24]. The data analysis will be managed by MAXQDA10 software.

#### Rigor and trustworthiness

In this study, Lincoln and Guba’s criteria (1994), including credibility, dependability, confirmability, and transferability, will be used to assess the rigor and trustworthiness of the collected data [25].

### The second phase: constructing a results-based logic model for a healthy public policy

In this phase of the research, the development of a results-based logic model for a healthy public policy is performed based on the findings of the first phase of the study, interviews with key informants and a review of the results of literature in this field.

A logic model is a visual representation of a program that maps the relationships between invested resources, ongoing activities, and direct as well as long-term outcomes of program activities [23].

The program logic model (PLM) has been used in reproductive health planning [26–28]. Both quantitative and qualitative research are appropriate for presenting the health program. However, the qualitative approach is more valuable for designing health programs because it provides information based on the experiences, values, and perspectives of the participants and is not affected by preprepared quantitative categories [29]. The results of studies with grounded theory methodology have often been used as qualitative research methods in health care planning [29, 30]. Due to the provision of deep information based on the context and the relative coordination of its implementation steps with the logical model program, ground theory has turned it into a suitable and optimal source for designing and compiling a program logical model [21].

Examples of interview questions with key informants for developing the program are as follows:

- What can be done to prevent the tendency of women to use these techniques?
- In your opinion, what topics should the designed program include to be effective?
- What organizations and at what level should be involved in the designed program?
- Which organizations at the micro and macro levels should be in charge of these programs?
- Regarding these women, what important points should be considered that have been neglected?

### The third stage: validation of the designed program

In this phase, a nominal group technique (NGT) will be used to validate the designed program in the presence of a group of experts [22, 31]. This technique is an important method for validating programs [32]. This method has been proposed as a valuable tool to facilitate group decision-making to make decisions and priorities, especially in the health system [33]. One of the advantages of this technique is that its results are determined at the end of a session, and it is possible to save time and provide immediate feedback [34]. In addition, in this method, it is not possible to influence a powerful person and induce their opinion [35].

To validate the program, a draft of the designed program will be provided to the key informants before the meeting to obtain deep insight into the program. The duration of a nominal group meeting is variable and depends on the size of the group, the number of questions and the type of participants.

According to the NGT, the meeting will be held according to the following steps:

Introductory statement, silent generation of ideas, choosing and prioritizing individual top lists, time out and icebreaker, discussion of group top issues, reranking and rating group top ideas, conclusion.

## Discussion

The present study will explore the decision-making process of FGCPs and will later design and validate a results-based logic model for a healthy public policy to reduce or prevent these procedures. In the review of literature, most of the studies conducted in this area were performed in Western countries, were performed quantitatively, and were guided by the paradigm of positivism [16–18]. For example, in a retrospective study of 163 French women who underwent labiaplasty, the motivations reported by women for performing this procedure were as follows: dissatisfaction with the appearance of the labia (87%), discomfort when wearing clothes (64%), discomfort when participating in sports (26%) and painful sexual intercourse (43%) [3].

There are limited qualitative studies in this field [17–19, 36]. Moreover, a deep understanding of why and how complex phenomena such as the abovementioned issue can be achieved through qualitative studies. In this regard, Locatelli et al. (2017) conducted a qualitative study with a grounded theory approach to explain the decision-making process to perform cosmetic surgeries (including general and genital cosmetic surgeries). Based on the results of the in-depth interviews, the two main factors identified that can lead people to decide to undergo cosmetic surgery include recent life events and their psychological needs [19].

Sharp et al. [16], in a content analysis study, explored the reasons for doing labiaplasty and the expectations and experiences of Australian women. In this study, the emerging themes included “media influence”, “negative experiences and interpretations”, “physical reasons versus reasons for beautification”, “satisfaction with surgery” and “sexual health”. In other words, online media’s representation of genital appearance and past negative experiences, especially in sexual matters, led women to worry about their sexual appearance. In addition, issues related to physical discomfort were also commonly raised, and sometimes women emphasized it to legitimize surgery. Most women were generally satisfied with the results of their surgery, although some noted that their labia were not as small or symmetrical as they had hoped. Most women reported a significant improvement in their sexual health after surgery; however, some noted that their emotional distress during sex did not improve [18].

In a qualitative content analysis study with the participation of 9 women undergoing cosmetic genital surgery in the city of Erbil, Iraq, Pirro et al. (2022) explored women’s understanding of and experience performing cosmetic surgery. Based on the findings of this study, genital surgeries lead to improvements in body image, sexual performance and sexual satisfaction in couples, especially spouses [36].

In a qualitative study conducted by Bramwell et al. (2007) on six British women undergoing labiaplasty, all women reported abnormal genital appearance and stated that they were seeking a normal genital appearance through labiaplasty. They also reported that they received confusing messages about whether the genitals were normal and whether surgery was necessary when consulting health practitioners. Finally, all of them reported that the inappropriate appearance of their genitals hurts their sexual relationships. Finally, the results of this study showed that labiaplasty does not necessarily improve the quality of sexual life of all people [17].

As noted, the current literature review shows that most of the studies have focused on the reasons for and consequences of performing FGCPs rather than the decision-making process of women.

The findings of the present study are expected to present a theory based on the cultural and social context of Iran to explain the decision-making process of this group of women, considering the purpose of ground theory, which is to study social phenomena embedded in human interactions.

According to this theory, in addition to individual and cognitive factors, structural factors such as interpersonal and social factors, situations that put people under pressure, and structural factors such as physical, social, and cultural issues are expected to be used to design suitable interventions for reducing the demand for these techniques.

In the second part of the study, the researchers will try to provide a results-based logic model for a healthy public policy to reduce or prevent these procedures by understanding the decision-making process of women and using the points of view of key informants. The logical model is one of the successful tools for planning in health fields [22]. For example, this model has been used in the design of programs to prevent HIV, sexual violence, domestic violence, homelessness in drug addicts and obesity in teenagers [26–28, 37–39]. Regarding the application of FGCPs, preventive measures have been sporadically recommended by different organizations. For example, not using nonscientific words for advertising by professionals, counselling women to determine the root of the problem and providing a specific solution and identifying women with psychological disorders or sexual dysfunction are among these suggestions [40–42]. However, a comperhensive program that is focused on the various dimensions of issues faced by these women during the long-term career decision process is not available.

Therefore, in this research, the researchers will present a program that is suitable for policymakers, planners and providers of healthcare services to be implemented in the social-cultural context of Iran. Using the designed program, healthcare practitioners will be able to provide more effective advice and guidance to women to make correct and informed decisions.

In the present study, the researchers predict several challenges. For example, women may be less willing to talk about their private and sexual issues. In addition, some of these women are not married and have sexual partners, which is considered taboo in the cultural and social context of Iran. Therefore, the importance of honesty in answering questions and gaining the participants’ trust in the confidentiality of the data will be emphasized. In addition, due to the low frequency of some procedures, such as G-spot augmentation, access to these women is limited. Therefore, with extensive advertising, it was possible to enter participants performing fewer common procedures to study. The present study, similar to other qualitative approaches, has limited generalizability, and the above limitations should be adjusted as much as possible by maximum variation in sampling.

## Data Availability

No datasets were generated or analysed during the current study.

## Abbreviations

FGCPs: Female genital cosmetic procedures
